# Storage and Diffusion of Carbon Dioxide in the Metal Organic Framework MOF-5—A Semi-empirical Molecular Dynamics Study

**DOI:** 10.1021/acs.jpcb.3c04155

**Published:** 2023-10-19

**Authors:** Risnita Vicky Listyarini, Jakob Gamper, Thomas S. Hofer

**Affiliations:** † Theoretical Chemistry Division, Institute of General, Inorganic and Theoretical Chemistry, University of Innsbruck, Innrain 80-82A, A-6020 Innsbruck, Austria; ‡ Chemistry Education Study Program, Sanata Dharma University, Yogyakarta 55282, Indonesia

## Abstract

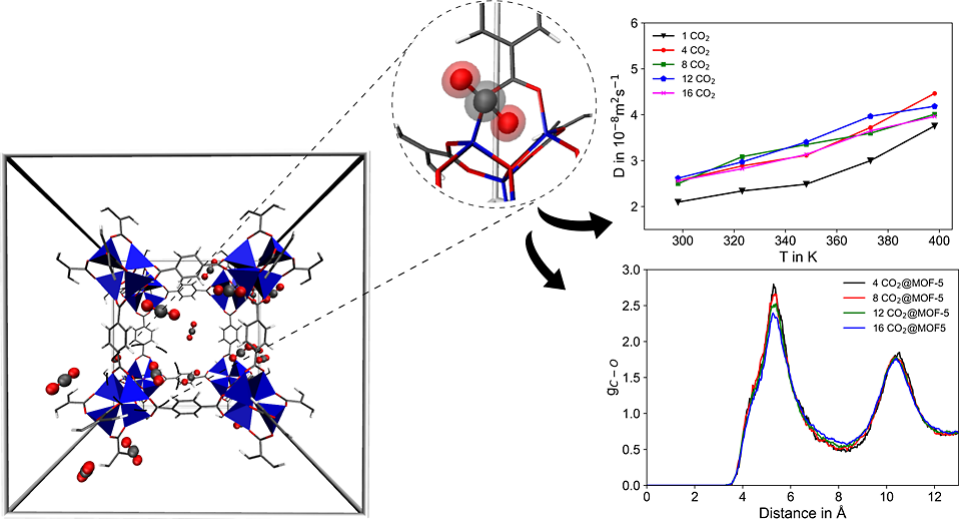

Metal–organic frameworks (MOFs) have attracted increasing attention due to their high porosity for exceptional gas storage applications. MOF-5 belongs to the family of isoreticular MOFs (IRMOFs) and consists of Zn_4_O^6+^ clusters linked by 1,4-benzenedicarboxylate. Due to the large number of atoms in the unit cell, molecular dynamics simulation based on density functional theory has proved to be too demanding, while force field models are often inadequate to model complex host–guest interactions. To overcome this limitation, an alternative semi-empirical approach using a set of approximations and extensive parametrization of interactions called density functional tight binding (DFTB) was applied in this work to study CO_2_ in the MOF-5 host. Calculations of pristine MOF-5 yield very good agreement with experimental data in terms of X-ray diffraction patterns as well as mechanical properties, such as the negative thermal expansion coefficient and the bulk modulus. In addition, different loadings of CO_2_ were introduced, and the associated self-diffusion coefficients and activation energies were investigated. The results show very good agreement with those of other experimental and theoretical investigations. This study provides detailed insights into the capability of semi-empirical DFTB-based molecular dynamics simulations of these challenging guest@host systems. Based on the comparison of the guest–guest pair distributions observed inside the MOF host and the corresponding gas-phase reference, a liquid-like structure of CO_2_ can be deduced upon storage in the host material.

## 1 Introduction

Greenhouse gases are the primary factors of climate change,^1^ with carbon dioxide (CO_2_) being by far the foremost crucial factor for global warming. The global average temperature has increased by more than 1 °C since 1850,^1^ and it is expected to reach 1.5 °C between 2030 and 2052 if the current emission rates remain. During the last half-century, the CO_2_ concentration increased to over 380 ppm and is expected to reach 550 ppm in 2050.^2^ Massive CO_2_ emissions into the atmosphere are primarily driven by higher energy demands.^3^ Fossil fuel-based CO_2_ emissions contributed to approximately 74% of the global greenhouse gas emission in 2018.^2^

Numerous efforts have been made to reduce the level of CO_2_ in the atmosphere. Capture of CO_2_ from the atmosphere and subsequent activation are considered as promising solutions to reduce its overall concentration. Postcombustion CO_2_ capture^4^ associated with CO_2_ separation from flue gas in industrial processes typically involves energetically highly intensive, costly, and inefficient chemical absorption, e.g., via aqueous amine-based solutions.^4−7^ Thus, energetically more efficient and technically feasible CO_2_ capture techniques employing porous materials have emerged. In the last two decades, novel porous materials referred to as metal–organic frameworks (MOFs) have attracted increasing attention due to their manifold properties.^8^ MOFs are crystalline porous compounds composed of secondary building units (SBUs) or nodes and organic linkers. The variety of SBUs (e.g., metal ions or metal-oxide clusters), organic linkers, and their various connectivities lead to near-endless possibilities in the design of MOF materials. The high internal surface areas and tunable porosities contribute to the properties, e.g., in gas storage, and separation applications,^9^ while their metal-containing nodes may provide active sites relevant for catalysis.^10^ MOFs can accommodate different types of guests, e.g., gases,^11−14^ ions,^15^ nanoparticles,^16,17^ and biological chemicals.^18^ As a result, MOFs have extensive applications, for example, in gas storage,^19^ gas separation,^20^ catalysis,^21,22^ luminescence,^23,24^ electrochemical applications,^25^ and drug delivery.^26,27^

Initially, MOFs became well-known for their excellent energy-relevant gas storage applications (e.g., H_2_, CH_4_, and CO_2_ storage^5,11−14^) being the result of their high porosity and surface area.^9,28,29^ To capture CO_2_ efficiently, MOFs should have unique characteristics and prerequisites, e.g., porosity, thermal stability, functional groups in the pores that can interact with CO_2_ that improve adsorption, the existence of open metal sites, and reversible adsorption and desorption behavior.^30^ One of the earliest MOFs displaying notable H_2_ gas storage capabilities^31,32^ is MOF-5, also known as IRMOF-1.^33−37^ MOF-5 belongs to the family of isoreticular MOFs (IRMOFs) that consist of Zn_4_O^6+^ clusters linked by 1,4-benzenedicarboxylate (BDC) units to form a cubic lattice. In addition to pronounced H_2_ storage capacity, it was shown that MOF-5 can also adsorb an unprecedented amount of CO_2_ in its cages.^38−40^ A recent review article by Sun et al.^41^ highlights new trends in MOF-based CO_2_ storage/trapping methods and novel synthetic strategies to further improve the storage capacities of these materials.

Understanding the properties of pristine MOF-5 as well as its host–guest interaction is therefore crucial for developing its practical applications. Experimental work on CO_2_ storage often requires enormous cost and time.^42^ Computational approaches can be employed as an alternative strategy to investigate host–guest interactions in MOF carriers. The grand canonical Monte Carlo method and molecular dynamics (MD) are widely employed to study the properties of the gas@MOF systems, e.g., argon, N_2_,^43,44^ H_2_,^44,45^ CH_4_,^7,44,46^ and CO_2_.^7,44^ While density functional theory (DFT) provides a high-level description of molecular interactions, its computational effort proves too demanding when considering the large number of atoms in the unit cell of MOFs.^47^ However, more efficient empirical potential models (i.e., pairwise-additive chemical force fields, FF) are often not sufficiently accurate to model the challenging host–guest interactions.^48^ To overcome this limitation, an alternative semi-empirical approach using a set of approximations and extensive parametrization of interactions known as density functional tight binding (DFTB)^49,50^ in combination with a suitable MD protocol has been employed. DFTB has shown an exceptional performance in the description of different MOF as well as guest@MOF system^51−55^ in the past.

The properties of pristine MOF-5, namely, its structure, the thermal expansion coefficient α, and the bulk modulus *K*, as well as the properties of adsorbed CO_2_, have been investigated in this work. Different loadings of CO_2_ have been introduced, and the associated self-diffusion coefficient *D*_s_ as well as the activation energy *E*_a_ have been investigated. Moreover, a series of host–guest and guest–guest pair distribution functions have been analyzed. This study provides detailed insight into the capability of the semi-empirical DFTB approach in MD simulation studies of these complex guest@host systems.

## 2 Methods

In this study, the semi-empirical DFTB method^49,50,56−59^ has been applied. DFTB is a DFT-based parametrization of tight-binding models frequently used in the treatment of solid-state systems.^60,61^ The iterative self-consistent charge (SCC)^62^ extension of DFTB was developed considering charge self-consistency^63^ based on a second-order Taylor series expansion (DFTB2) with respect to the associated reference charge density ρ^0^.^64^ The respective energy *E*^SCC-DFTB^ consists of three terms described as follows.
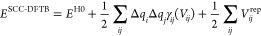
1

The first term is the sum of energy contributions from an atomic orbital Hamiltonian depending on the associated reference density *E*^H0^.^64^ The second term accounts for charge-transfer contributions in which the function γ_*ij*_ modulates the associated charge–charge interactions. At a large distance, *r*_*ij*_, γ_*ij*_ reduces to the well-known 1/*r*_*ij*_ form that describes the Coulomb interaction of the partial charges Δ*q*_*i*_ and Δ*q*_*j*_. In the case of *i* = *j*, the Hubbard *U*_*i*_ parameter is introduced to describe charge transfer to/from a particular atom. The latter is related to the chemical hardness, taking the second derivative of the energy with respect to charge density into account. The third term represents the sum of pairwise repulsive potentials, *V*_*ij*_^rep^. In addition, the inclusion of a third-order term of the Taylor series expansion, requiring the so-called Hubbard derivatives to describe the charge dependence of the chemical hardness of the individual atoms, is referred to as DFTB3.

### 2.1 Simulation Protocol

The starting structure of MOF-5 was taken from an earlier simulation study.^54^ The initial lattice parameters of the cubic simulation cell amount to 26.11828 Å, and the fixed associated lattice angles α, β, and γ remained fixed at 90°. All simulations were performed using third-order SCC DFTB^49,64^ calculations under periodic boundary conditions using the third-order parameter set for organic and biological applications (3ob).^65,66^ In order to assess the performance of dispersion correction, the D3^67^ (with Becke and Johnson damping, BJ)^68^ and D4^69^ dispersion correction was included. As was previously shown^54^, the application of Γ-point sampling in the integration of the Brillouin zone^70^ is sufficient for MOF-5 due to the larger size of the unit cell. The DFTB MD simulations were performed using an in-house developed QM/MM MD^71−73^ simulation program interfaced to the DFTB+ package.^74^

The velocity-Verlet algorithm with a time step of 2.0 fs was employed to integrate the equations of motion.^75^ The simulations were performed with all bonds involving hydrogen atoms being constrained via the SHAKE/RATTLE algorithms.^76,77^ The respective constraint distances had been derived from preliminary MD simulations using a shorter MD time step of 0.5 fs, enabling full flexibility of all bonds in the system.^54^ An isothermal–isobaric (*NPT*) ensemble was achieved using a Nose–Hoover chain thermostat (chain length of 5) and Berendsen manostat algorithm^78^ with a relaxation time of 5.0 ps. The target temperatures and pressure were set in the range from 248.15 to 348.15 K in 25 K increments and at 1.013 bar, respectively. The equilibration was performed for 5000 MD steps (10 ps), and data sampling for 160,000 MD steps (320 ps).

In order to assess the response of MOF-5 to external pressure, the system was also treated under *NVT* conditions at 298 K. A series of constant volume and temperature (*NVT*)^79^ simulations were carried out considering variations of ±0.1% from the *NPT* equilibrated volume *V*_eq_. The equilibration was performed for at least 10,000 MD steps (20 ps), followed by data sampling for 60,000 MD steps (120 ps).

The interaction energy of the MOF-5 host and a single CO_2_ guest molecule was evaluated based on MD simulations under *NPT* conditions based on reference simulations of the isolated MOF-5 and CO_2_ systems at 298.15 K. Equilibration for the isolated systems was performed for 5000 MD steps (10 ps), followed by 50,000 MD steps (100 ps) of sampling.

The presence of CO_2_ guest molecules in the host MOF-5 was investigated at different amounts of loading. Loading variations of 1, 4, 8, 12, and 16 CO_2_ molecules in MOF-5 were considered in this study. The target temperature and pressure were set from 298.15 to 398.15 K with increments of 25 K and to 1.013 bar, respectively. The equilibration was performed for at least 25,000 MD steps (50 ps) followed by data sampling for a total of 265,000 MD steps (530 ps).

### 2.2 Data Analysis

#### 2.2.1 Powder X-ray Diffraction Pattern

The powder X-ray diffraction (PXRD) patterns of rigid MOF-5 were analyzed for the optimized structure obtained via energy minimization, as well as by averaging a series of individual PXRD patterns determined from the SCC DFTB MD trajectory. The associated trajectory was converted into the corresponding crystallographic file (CIF) for every fifth configuration and then used as input into RIETAN-FP^80^, provided by VESTA.^81,82^ A total of 15,500 individual PXRD patterns obtained from the simulation trajectory have been averaged. The most prominent reflection of each diffractogram was then normalized to an intensity of 100 for visualization and comparison purposes. The respective PXRD obtained from the DFTB MD trajectory and via energy minimization had been compared with the experimental reference data taken from reference Purtscher et al.^54^, obtained at Mo Kα radiation (λ = 0.709319 nm).

#### 2.2.2 Linear and Volumetric Thermal Expansion Coefficient of MOF-5

The linear thermal expansion coefficient α_*a*_ is defined as the relative change in lattice parameter with respect to temperature at a constant pressure.
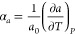
2

The linear thermal expansion coefficient of MOF-5 at 298 K was determined as follows
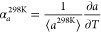
3

In this work, a total of four neighboring temperature points were employed to determine the respective slope via finite differentiation.^55^
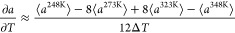
4

The volumetric thermal expansion coefficient α_*V*_ was calculated in a similar fashion
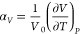
5
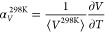
6
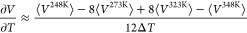
7

#### 2.2.3 Bulk Modulus of MOF-5

The response of MOF-5 to compression can be studied from its bulk modulus, which was obtained as follows,
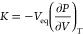
8

A series of constant volume^79^ simulations (i.e., *NVT* ensemble) employing the equilibrium volume *V*_eq_ (determined from the *NPT* simulation at 298 K and 1.013 bar) as well as at variations in *V*_eq_ by ±0.1% have been carried out to determine the respective average pressure. The associated gradient in the pressure was then calculated via finite differentiation
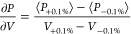
9with ⟨*P* ± 0.1%⟩ and *V*_0.1%_ being the respective average pressure and the volume of the simulations executed above and below the equilibrium volume.

#### 2.2.4 Determination of Interaction Energy

The instantaneous interaction energy *U*_int_ between the MOF-5 host and a CO_2_ molecule was determined as follows
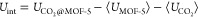
10 is the total energy (i.e., kinetic and potential energy) of the guest@host system while *U*_MOF-5_ and  correspond to the respective average total energies of the isolated MOF-5 and CO_2_ systems, respectively. ⟨*U*_MOF-5_⟩ and  were determined over the last 0.25 ns of the simulation trajectory. While in this approach, all variations in the geometry of the host–guest systems, including variations in the lattice parameters, are still visible in the time evolution of *U*_int_, an analysis of the respective running average employing 500 data points enables a visual inspection of the equilibration period as well as changes in *U*_int_ due to variations in the host–guest interaction.^55^

#### 2.2.5 Determination of the Self-Diffusion Coefficient of CO_2_ in MOF-5

The self-diffusion coefficient *D*_s_ represents the translational motion of CO_2_ in MOF-5. In a three-dimensional system, *D*_s_ can be determined from the mean square displacement (MSD)^83^ of CO_2_ after time *t* using the Einstein^83^ relation.^11,44,84−86^
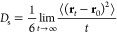
11**r**_*t*_ and **r**_0_ are the positions of CO_2_ at time *t* and the time origin *t*_0_, respectively. A running correlation window of 10 ps (2500 MD configurations) was employed in the determination of the MSD considering the last 500 points for the linear fit to obtain the respective *D*_s_ value.

The associated activation energy *E*_a_ was determined by using a linear fit to the Arrhenius equation^87^
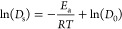
12with *D*_0_ and *R* being the pre-exponential factor and the molar gas constant, respectively.

### 2.3 Radial Distribution Functions

The interaction between the CO_2_ guest molecules and MOF-5 was evaluated using radial distribution functions (RDFs) based on the C–C interatomic distances of the CO_2_–CO_2_ molecule pairs as well as C–O interatomic pairs of the CO_2_ and Zn_4_O^6+^ clusters of MOF-5.

## 3 Results and Discussion

In the following, the results of the SCC DFTB MD study of pristine MOF-5 as well as its host–guest interaction as a function of an increasing number of CO_2_ molecules are presented. The structural properties of MOF-5 are evaluated from the respective PXRD analysis. The negative thermal expansion (NTE) behavior is investigated based on the associated lattice parameter *a*, the linear and volumetric thermal expansion coefficients α_*a*_ and α_*V*_ as well as the bulk modulus *K*. Detailed insights into the dynamic properties of the host–guest interaction for the case of a single CO_2_ molecule as well as guest–guest interactions at higher loadings of CO_2_ molecules were evaluated from the associated interaction energy *U*_int_, self-diffusion coefficients *D*_s_, activation energies *E*_a_, and the respective RDFs. The total accumulated simulation time of all MD runs carried out in this study amounts to 17 ns.

### 3.1 Properties of Pristine MOF-5

#### 3.1.1 X-ray Diffraction Patterns

The structural analysis of pristine MOF-5 is carried out using the simulated PXRD patterns obtained for the respective energy-minimized structure, also considering the cell-parameters (i.e., 0 K condition), as well as via averaging over PXRD patterns obtained for more than 15,000 configurations extracted from the MD trajectories at regular intervals^54^ (see Figure S1). In addition, the inclusion of dispersion correction employing the D3 model^67^ with Becke and Johnson damping^68^ and the newer yet more demanding D4 approach^69^ was evaluated.

The X-ray diffractograms of MOF-5 obtained via averaging over the MD trajectory at SCC DFTB/3ob/D3 and D4 level are shown in Figure S1 and appear similar to the experimental reference data^54^ and those obtained via energy minimization. The reflexes in the range from 5 to 20° show good agreement with the experimental reference taken from Purtscher et al.,^54^ in particular, the two prominent reflexes observed at 2θ of ∼6.23 and 6.96°. Since no significant difference between the diffractograms obtained using the D4 and D3 dispersion corrections can be detected, the latter level of theory was considered for the simulations in this study, as the D3 correction is computationally less demanding.

**Figure 1:**
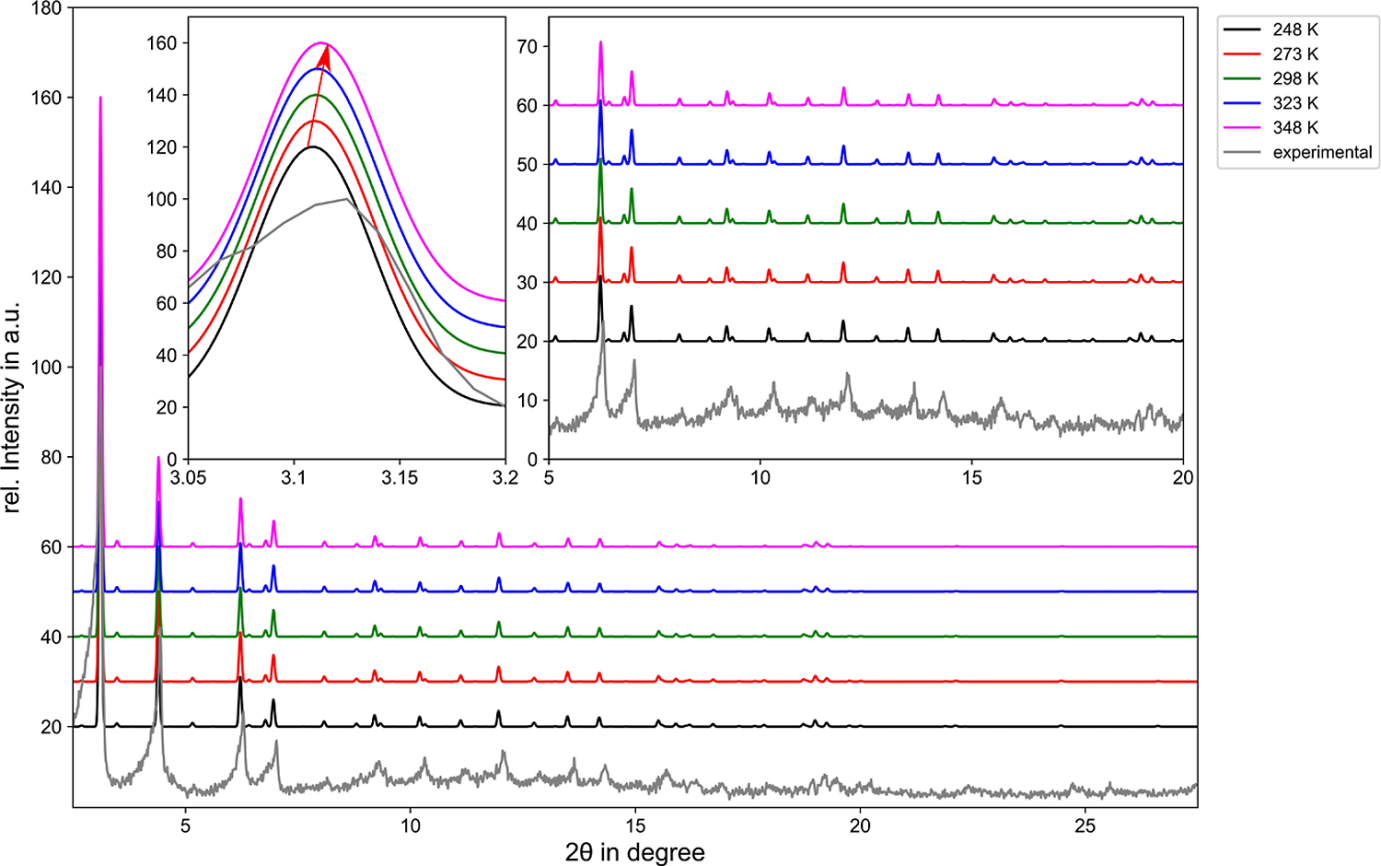
Comparison of X-ray diffractograms of MOF-5 obtained via averaging over the MD trajectories at SCC DFTB/3ob/D3 level at different temperatures and the experimental PXRD ref (54) (Mo Kα, λ = 0.709319 nm).

A comparison of X-ray diffractograms of MOF-5 in the temperature range of 248–348 K is depicted in Figure 1. The two prominent reflexes near ∼6.23 and 6.96° as well other reflexes in the range of 5–20° agree well with the experimental data.^54^ When comparing all investigated temperatures, a slight drift of the main reflex to higher angles occurs in the range of 3.1–3.15°, pointing toward a decrease in the size of the unit cell. No structural deformation of MOF-5 is observed at higher temperatures of up to 398 K, indicating that the nanoporous structure is maintained over the entire temperature range.

#### 3.1.2 Lattice Parameter and Thermal Expansion

MOF-5 has a cubic structure and is widely known to display an isotropic NTE, which has been proven both experimentally^88−91^ and theoretically.^84,92−96^ This isotropic NTE behavior is desirable in material design because the particle orientation does not affect expansion, making them an excellent choice in many technical applications^97^, such as electronic devices, mechanical machining, and as engineering materials.^98,99^

A comparison of the corresponding average lattice parameter of MOF-5 at 298 K obtained from the MD simulations to data from the literature is shown in Table 1. The calculated lattice parameter differs from the measured experimental value^88,90,100,101^ by approximately 1.01%. In particular, the corresponding value of 26.1353 Å agrees almost perfectly with the value of 26.088 Å obtained by using the B3LYP/DFT level of theory. Considering that the 3ob DFTB parametrization was designed with a focus on organic and bioorganic molecules,^65^ the fact that the 3ob parametrization can accurately reproduce the associated lattice parameter is indeed remarkable.

**Table 1:** Lattice Parameter of MOF-5 Obtained in This Work in Comparison to Other Simulated and Experimentally Determined Estimates^a^

methods	*T* (K)	*a* (Å)	references
SCC DFTB/3ob/D3 MD	298	26.135	this work
SCC DFTB/3ob/D3 avg	298	26.134	(54)
SCC DFTB/3ob/D3 opt	298	26.270	(54)
XRD	298	25.887	(100)
XRD	258	25.832	(101)
NPD	298	25.8	(88)
PXRD	299.7	25.821	(90)
MM opt		25.945	(119)
MM opt		26.08	(120)
LDA/DFT opt		25.888	(121)
GGA/DFT opt	298	25.77	(122)
B3LYP/DFT opt		26.088	(123)

a MD: molecular dynamics; avg: averaging MD trajectory; opt: optimization; NPD: neutron powder diffraction; (P)XRD: (powder) X-ray diffraction; FF: force field; DFT: density functional theory; LDA: Local-density approximation; GGA: generalized gradient approximations.

**Figure 2:**
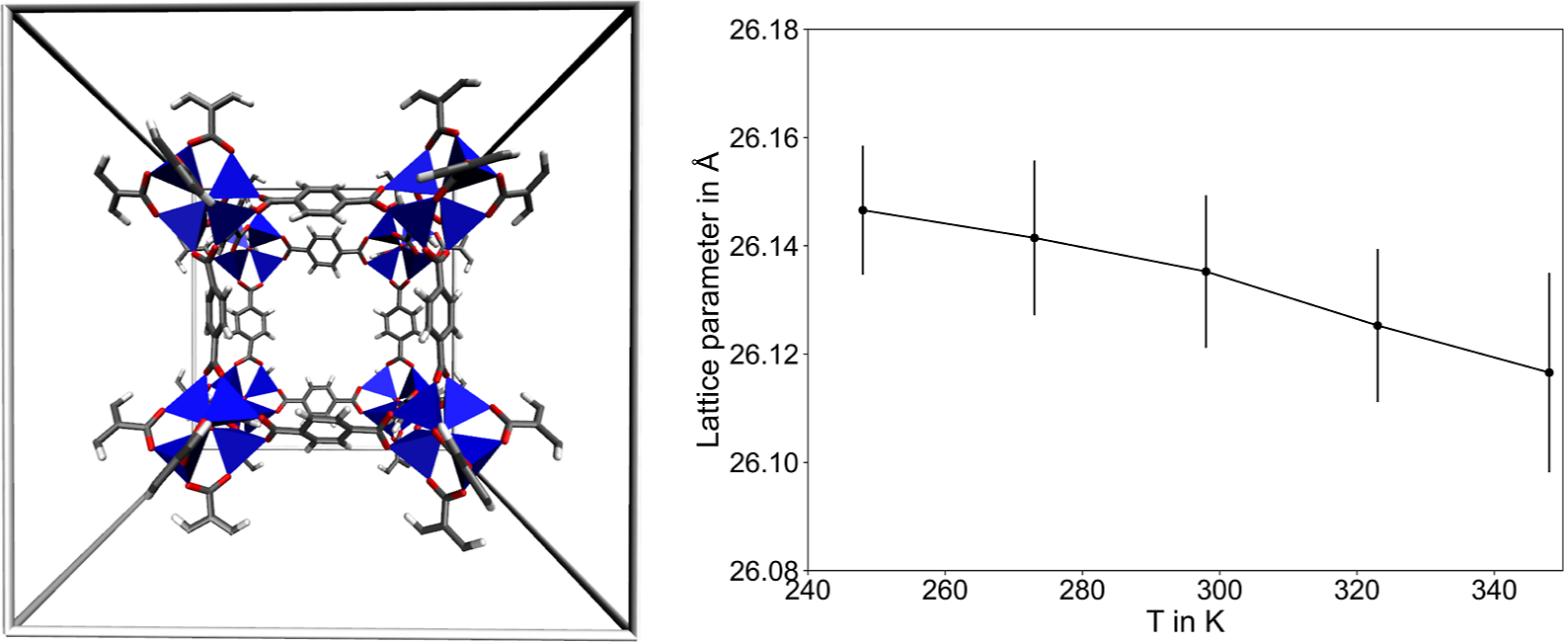
Left: Unit cell of the pristine MOF-5 simulation system. Right: Temperature dependence of the associated lattice parameter determined via SCC DFTB/3ob/D3 MD simulations indicating a negative thermal expansion.

The NTE behavior can be evaluated directly from the change in the average lattice parameter with respect to variations in temperature. The temperature dependence of the associated lattice parameter determined via SCC DFTB/3ob/D3 MD simulations is depicted in Figure 2. The average lattice parameter ranges between 26.1466 and 24.1166 Å for the temperature range of 248–348 K, respectively. At an elevated temperature of 348 K, the average lattice parameter decreases by approximately 7.76%, indicating that the size of the unit cell decreases notably with increasing temperature.

The linear and volumetric thermal expansion coefficients α_*a*_ and α_*V*_ have been determined via finite differentiation according to eqs 4 and 7 and are compared to theoretical and experimental estimates found in the literature in Table 2. The α_*a*_ value of −12.69 MK^–1^ determined for the temperature range of 248–348 K is in excellent agreement with the experimental and calculated values reported in the range of −17.6 to −10^88−91^ and −21.5 to −2.9 MK^–1^^84,92−96^, respectively. In addition, the volumetric thermal expansion coefficient α_*V*_ of −38.07 MK^–1^ is found in excellent agreement with other experimental and simulated data in the range of −30.4 to −41 MK^–1^.^84,92−94^ Based on these findings, it can be concluded that the SCC DFTB/3ob/D3 approach is able to accurately represent the NTE of MOF-5 in MD simulations.

**Table 2:** Thermal Expansion Coefficient of MOF-5 in Comparison with Other Experimental and Simulated References^a^

methods	α_*a*_ (MK^–1^)	α_*V*_ (MK^–1^)	*T* (K)	references
SCC DFTB/3ob-D3	–12.69	–38.07	248–348	this work
vacuum PXRD	–13.1	–39	80–500	(90)
vacuum PXRD	–14.5(1)		100–325	(91)
vacuum PND	–12.0		20–400	(91)
NPD	–16 to −10		4–600	(88)
MM MD	–10.01	–30.4	50–600	(84)
MM MD		–37	200–400	(92)
MM MD	–18	–40	100–1000	(94)
MM MD		–41	300	(84,93)
MM MD	–2.9 to −18.9		80–500	(95)
MM MD	–14.2 to −21.5		0–500	(96)
MM MD	–5.27	–15.80	80–500	(34)
LD (QHA; PBE)	–17.6		300	(88)
GG (QHA; B3LYP-D3)	–10.6		300	(89)

a NPD: neutron powder diffraction; (P)XRD: (powder) X-ray diffraction; LD: local density; GG: Generalized Gradient; QHA: quasi harmonic approximation.

#### 3.1.3 Bulk Modulus of MOF-5

The mechanical stability of MOF-5 is evaluated from the respective bulk modulus *K*. The latter is the inverse of the compressibility and represents the mechanical stability of a solid to resist compression deformation within the limits of elasticity.^97,102^ The bulk modulus is a crucial property for a material, especially for cubic crystals, to characterize its hardness.^97^ In this work, the bulk modulus was evaluated according to eq 8 by carrying out a series of simulations in the *NVT* ensemble employing the equilibrium volume *V*_eq_ determined in an *NPT* simulation at 298 K as well as at variations in *V*_eq_ by ±0.01%.

Table 3 summarizes the comparison of *K* with other simulation results reported in the literature. The corresponding value for *K* of 14.73 GPa obtained from the SCC DFTB/3ob/D3 MD simulation compares well with another simulated reference value of 15.34 GPa^52^ obtained using the DFTB method, as well as other MM and DFT studies reporting values in the range of 11.95–22 GPa. It should be noted that although the reference values are mostly derived using the Birch–Murnaghan equation of state, the corresponding estimates for *K* lie in between those obtained via MM models and higher-level DFT results.

**Table 3:** Comparison of the Bulk Modulus *K* of MOF-5 Obtained in This Work with Other References Using Different Simulation Methods and Equations^a^

methods	method to obtain *K*	*K* (GPa)	references
SCC DFTB/3ob MD	eq 8	14.73	this work
BTW-FF MD	BMEOS	11.95	(34)
BTW-FF opt	MEOS	13.6	(95)
UFF opt	MEOS	14.5	(95)
UFF4MOF opt	MEOS	16.8	(95)
DWES opt	MEOS	17.5	(95)
DREIDING opt	MEOS	22.0	(95)
DFTB opt	MEOS	15.34	(52)
DFT/GGA opt	MEOS	15.37	(124)
DFT/GGA opt	BMEOS	16.3	(125)
DFT/LDA opt	BMEOS	17.6	(125)
DFT/LDA opt	BEOS	17	(121)
DFT/LDA opt	BMEOS	17.9	(126)
DFT/LDA opt	fit	18.2	(127)
DFT/LDA opt	fit	18.5	(128)

a UFF: universal force field; UFF4MOF: extended UFF atom optimized for MOFs; DWES: Dubbeldam, Walton, Ellis, and Snurr; MD: molecular dynamics simulation; opt: optimized structure; BMEOS: Birch–Murnaghan equation of state; MEOS: Murnaghan equation of state; BEOS: Birch–Murnaghan equation of state; fit: by fitting the energy-volume dependency.

### 3.2 CO_2_@MOF-5

#### 3.2.1 Interaction Energy of MOF-5 and CO_2_

**Figure 3:**
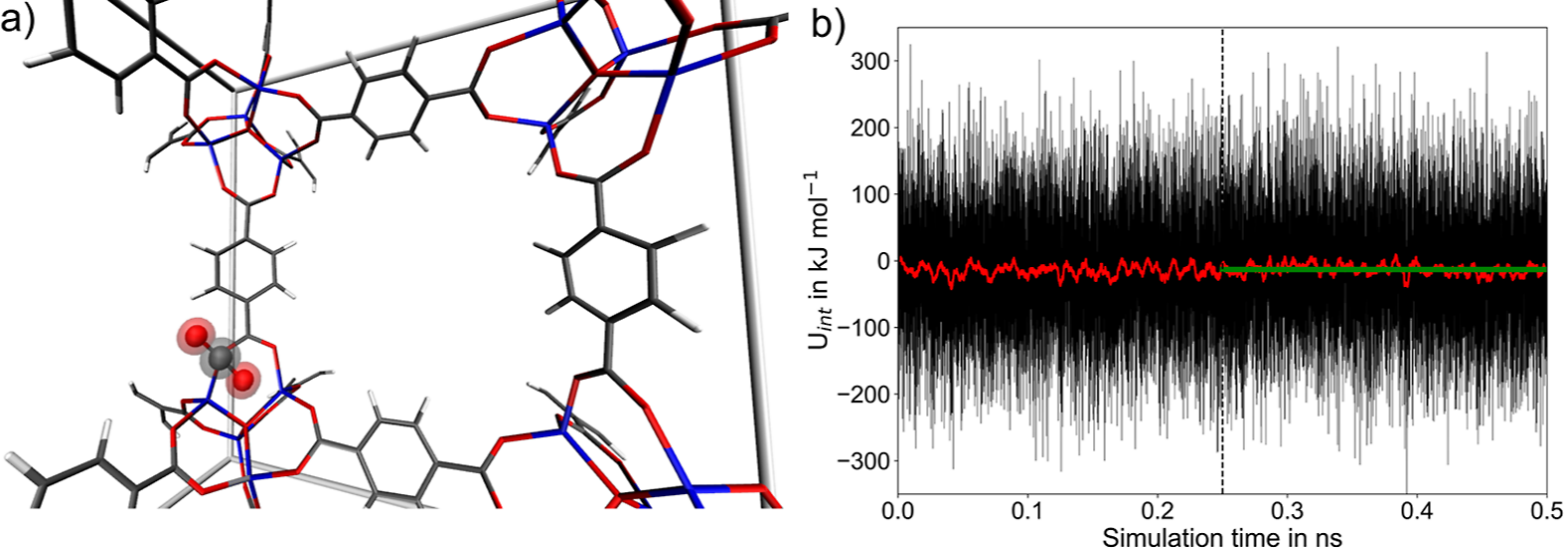
(a) Snapshot of the interaction motif of a single CO_2_ guest molecule with a Zn_4_O^6+^ SBU of MOF-5 observed in the SCC DFTB/3ob/D3 MD simulation. (b) Time evolution of the CO_2_-MOF-5 interaction energy (black) and the respective running average based on a window size of 500 data points. The associated average value (green) has been determined using only the last 0.25 ns of the simulation trajectory.

A snapshot of the reoccurring interaction pattern of a single CO_2_ molecule coordinated to a Zn_4_O^6+^ SBU of MOF-5, as well as the time evolution of the host–guest interaction energy *U*_int_, are depicted in Figure 3a,b. The latter was calculated from the simulation of the combined CO_2_@MOF-5 system. In addition, separate MD simulations of the isolated MOF-5 host and an isolated CO_2_ molecule have been carried out, yielding the respective average reference energies ⟨*U*_MOF-5_⟩ and . In order to monitor the time evolution of the host–guest interaction, the instantaneous values for *U*_int_ have been considered (see eq 10). The observed strong oscillations in the time evolution of *U*_int_ arise due to the structural changes in the CO_2_@MOF-5 system, in particular variations in the host–guest interaction, structural changes in the linker and inorganic units, as well as variations of the unit cell due to constant pressure conditions. Especially the latter are known to have a strong impact on the instantaneous interaction energy at the short time scales that quickly average, as can be seen from the associated running average also included in Figure 3b.

The average ⟨*U*_int_⟩ value of −13.0 kJ mol^–1^ determined over the second half of the simulation compares very well with experimental results^103^ for the heat of adsorption of −15.1 and −14.9 kJ mol^–1^ and other theoretical data^104^ reported as −9.27 and −14.0 kJ mol^–1^, respectively.

#### 3.2.2 Diffusion Analysis

**Figure 4:**
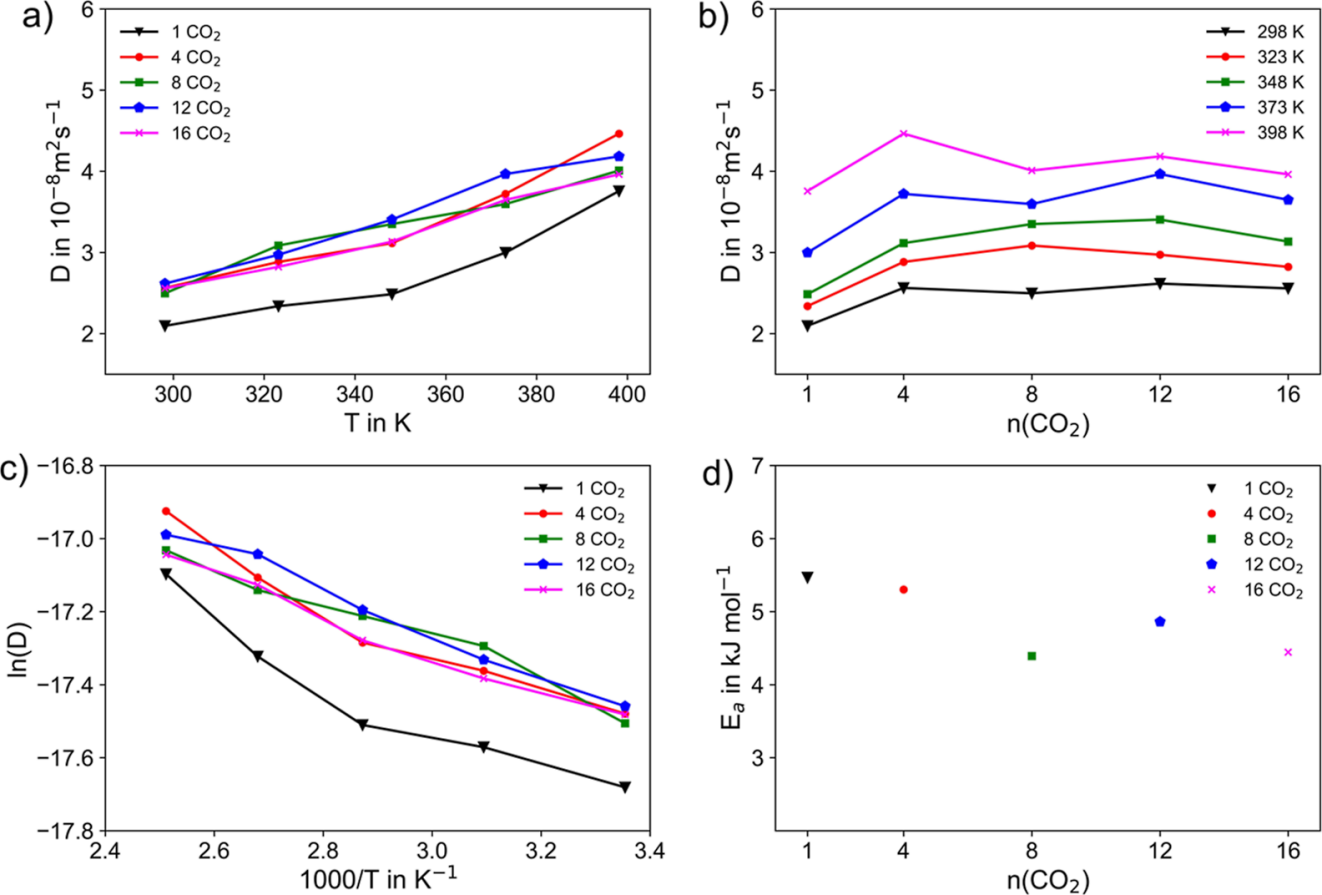
(a,b) Self-diffusion coefficient of CO_2_ for different loadings and temperatures (c) Arrhenius representation of the self-diffusion coefficient in m^2^ s^–1^ and (d) associated activation energies *E*_a_, as a function of CO_2_ loadings.

The diffusive properties of the CO_2_ molecules embedded in the MOF-5 host can be directly obtained from MD simulations via the average MSD.^83^ The observed self-diffusion coefficients of CO_2_ in the MOF-5 host are displayed in Figure 4a,b. as a function of temperature and loading, the corresponding Arrhenius representation is shown in Figure 4c. The respective plots of the MSD against a correlation time of 10 ps are exemplary for a single CO_2_ molecule embedded in MOF-5 in Figure S2. An almost ideal linear dependence of the MSD in the long-time limit is observed for all temperatures, that is well separated from the associated ballistic regime.

In this study, five evenly spaced temperatures in the range from 298 to 398 K have been considered, which are well within the range of thermal stability of MOF-5 reported as up to 723 K.^105−108^ Similarly, five different loadings of 1, 4, 8, 12, and 16 CO_2_ molecules have been considered. For each system, 50 ps of equilibration followed by 0.53 ns for sampling has been carried out per temperature point, thus yielding a total accumulated simulation time of 14.5 ns.

The respective self-diffusion coefficient of 2.09 × 10^–8^ m^2^ s^–1^ agrees well with other simulated references values^7,44^ of 3.01 × 10^–8^ and 1.4 × 10^–8^ m^2^ s^–1^, respectively. However, these values deviate by several orders of magnitude when compared to experimentally measured values reported as 8.1 to 11.5 × 10^–9^ cm^2^ s^–1^.^109^ Nevertheless, a recent experimental study by Thissen et al.^110^ of fluorescein in the MOF HKUST-1 showed that the elimination of domain boundaries in the solid achieved by epitaxial growth of the MOF on a Si(111) substrate, increased the diffusivity of the organic guest molecule by more than one order of magnitude. The authors of this study concluded that macroscopic diffusion properties may be strongly affected by either boundaries between differently oriented grains or/and barriers associated with surface diffusion. On the contrary, the solid is treated as an infinite and ideal system in a simulation setup; hence, effects associated with surfaces and grain boundaries cannot be represented in an MD study employing a single, periodic unit cell.

The dependence of *D*_s_ with respect to CO_2_ loading as a function of temperature is depicted in Figure 4b. For all temperatures, a notable increase of the diffusion is observed when increasing the amount of loading from one to four CO_2_ molecules. A similar behavior was reported in a study of CO_2_ diffusion in the covalent organic frameworks HEX-COF1 and 3D-HNU5^111^, as well as for other small guest molecules embedded in a host matrix, e.g. CH_4_ and H_2_ in zeolite ZK4^112,113^ and CH_4_, CF_4_, He, Ne, Ar, Xe, and SF_6_ in silicalite.^114^ As soon as more than a single guest molecule is present inside the hosts, cooperative effects influencing the diffusive properties are observed. Skoulidas and Sholl^114^ studied light gases adsorbed as single components in silicalite and suggested that the decrease in *D*_s_ as a function of loading is mainly due to the steric interactions between gas molecules. In a related study of CH_4_ and H_2_ in zeolite ZK4, Tunca and Ford^112,113^ explained that this behavior occurs because of collective effects of molecules in neighboring cages that reduce the energy barrier for molecules to hop between cages relative to the energy barrier encountered by a single molecule. On the other hand, the steric hindrance at high loading will counteract this energy barrier and decrease the self-diffusivity due to crowding effects. These findings of a CO_2_ loading dependence are consistent with the studies of CO_2_ molecules embedded in MOF host materials,^7,44,115,116^ also reporting a monotonically decrease of *D*_s_ upon increased loading. The reduction of the cage-to-cage energy barrier can be associated with guest–guest (i.e., CO_2_–CO_2_) interactions. In general, a slow decrease of *D*_*s*_ is observed for all considered CO_2_ loadings in MOF-5 (see Figure 4d), as also observed in a computational study of Babarao and Jiang.^7^

The activation energy *E*_a_ for the diffusion of a single CO_2_ molecule has been determined from the SCC DFTB/3ob/D3 simulations as 5.47 kJ mol^–1^. This result lies in between the value of 4.05 kJ mol^–1^^7^ determined from MD simulations and the experimental reference of 7.61 kJ mol^–1^.^109^ The decrease in *E*_a_ upon higher loadings of CO_2_ observed in Figure 4d. suggests that the adsorption of additional CO_2_ inside MOF-5 is favorable. For smaller loadings of CO_2_, a larger number of vacant binding sites is available, which overall promotes host–guest interaction. This can be expected to also increase the reaction barrier associated to migration between the individual interaction sites. On the other hand, upon increase of the amount of loading, a competition of the molecules for the binding sites will arise, ultimately promoting the occurrence of diffusive events. Since the magnitude of the decrease in *D*_s_ depends on many factors such as pore volume, pore size, surface area, as well as the nature of the guest molecule, as also discussed by Babarao and Jiang,^7^ further simulations of similar systems (e.g., IRMOF-10^101,117^ and UMCM-9^118^) are required to derive a general trend.

#### 3.2.3 Motif of CO_2_@MOF-5 Interaction

**Figure 5:**
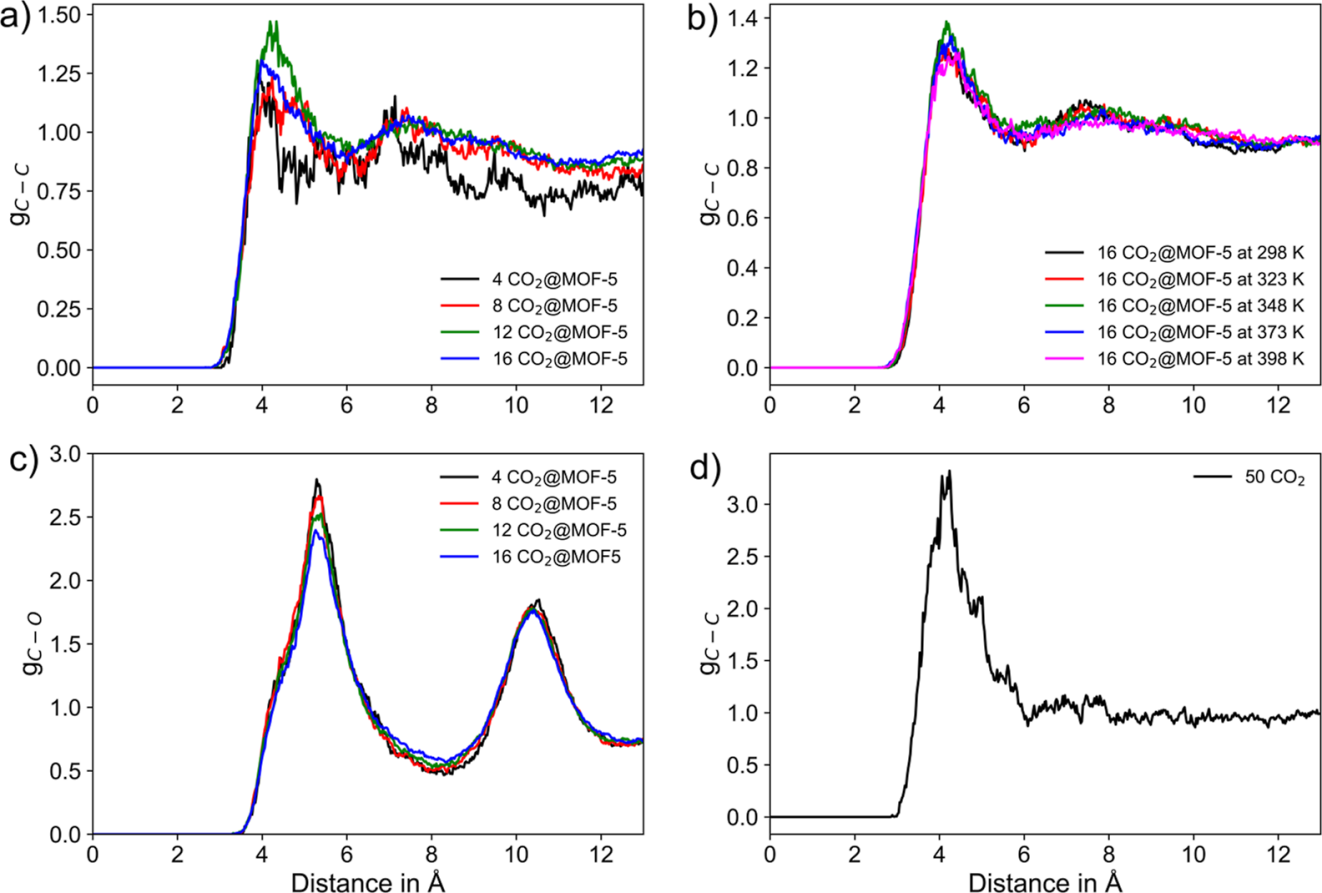
(a) RDF of the  pairs as the function of CO_2_ loading, (b) CO_2_–CO_2_ pairs in case of 16 CO_2_ loading as a function of temperature, and (c) C–O interatomic pairs of CO_2_ and Zn_4_O^6+^ SBU cluster of MOF-5.

In order to elucidate the guest–guest and host–guest interaction, RDF plots are constructed focusing on the C–C interatomic pair distance of CO_2_ molecules as well as the O atom of the Zn_4_O^6+^ SBU cluster of MOF-5 to the central C atom of CO_2_. Figure 5a. depicts the corresponding  interatomic pairs determined for different loadings of CO_2_ at 298 K. The main peak is located between 4.0 to 4.25 Å, and a minimum is found in the range of 4.5–6.0 Å, indicating distinct first neighbor  interactions. Interestingly, a second peak is found in the range of 7.0–7.5 Å, pointing toward a second domain of interaction between the CO_2_ molecules. It is visible that there is a slight but notable increase in intensity in the first neighbor  distance in case of higher loadings (i.e., 8, 12, and 16 CO_2_ molecules, respectively).

Figure 5b. depicts the CO_2_–CO_2_ pairs in the case of the 16 CO_2_ loading as a function of the temperatures. In all cases, the main peak is found at 4.2 Å followed by minima near 6.0 Å. It should be noted that the intensities remain similar at different temperatures for the same amount of CO_2_ loading.

The associated  interatomic pairs obtained from a simulation of 50 CO_2_ molecules in the gaseous state is shown in Figure 5d. The comparison of Figure 5a,d shows that the interaction of CO_2_–CO_2_ pairs in MOF-5 does not resemble that in the pure gas, notably due to the absence of a second neighbor peak in the range of 7.0–7.5 Å. It can be concluded that the CO_2_–CO_2_ interaction in MOF-5 does not resemble a gas-like interaction but shows a higher degree of ordering that points toward the formation of a liquid-like state upon confinement in the MOF host.

In addition, the host–guest interaction is evaluated from the associated O_MOF_–C_CO_2__ RDFs depicted in Figure 5c. Two prominent peaks are found near 5.3 Å and in the range from 10.4 to 10.6 Å. The two peaks observed in O_MOF_–C_CO_2__ pair distribution are due to the interaction of O atoms in the Zn cluster of MOF-5 with multiple CO_2_ in the proximity. The higher intensity shown in Figure 5c than that shown in Figure 5a suggests that the guest–host CO_2_@MOF-5 interaction is stronger than the guest–guest CO_2_–CO_2_ interaction.

## 4 Conclusions

With the large number of atoms in the unit cell of MOFs, quantum chemical levels of theory such as DFT have proved to be much too demanding when aiming at the execution of nanosecond-scale MD simulations. On the other hand, more efficient MM methods are oftentimes not sufficiently accurate to describe the complex host–guest interactions. As an alternative, in this study, the properties of pristine MOF-5 as well as the respective host–guest interaction with CO_2_ molecules were investigated via semiempirical SCC DFTB/3ob/D3 MD simulations, reaching an accumulated simulation time of 17 ns.

The properties of pristine MOF-5 have been evaluated in terms of the associated PXRD patterns, the lattice parameter *a*, the linear and volumetric thermal expansion coefficient α_*a*_ and α_*V*_ as well as the bulk modulus *K*. The negative linear and volumetric thermal expansion coefficients were determined as −12.69 and −38.07 MK^–1^, respectively, in excellent agreement with other experimental and simulated estimates. Similarly, the respective value for *K* of 14.73 GPa compares well with data in the literature. It can be concluded that these key properties can be accurately described via the presented SCC DFTB/3ob/D3 MD simulation strategy.

The structural, dynamical, and thermodynamic properties of the CO_2_@MOF-5 host–guest system have been characterized via the respective interaction energy, self-diffusion coefficient, activation energy, and RDFs. The average value of the interaction energy of −13.0 kJ mol^–1^ compares well with other theoretical results. The self-diffusion coefficient and the associated activation energy for a single CO_2_ molecule in the MOF-5 host have been determined as 2.09 × 10^–8^ m^2^ s^–1^ and 5.47 kJ mol^–1^, respectively and agree well with other experimental and simulated references. As also highlighted in a number of previous simulation studies of different nanoporous compounds also in this work a notable increase in the diffusivity is observed for loadings considering more than one guest molecule.

Based on the RDFs it could be shown that the guest–guest interaction in the confinement of the MOF host displays a higher degree of ordering compared to a reference simulation of neat CO_2_ gas at the same conditions. Furthermore, the RDF plots indicate that the interaction of O_MOF_–C_CO_2__ is stronger than guest–guest  interaction.

Overall, all of the theoretical results agree well with other experimental and theoretical references. The data presented in this work provide clear evidence that the applied SCC DFTB MD simulation strategy provides a versatile and efficient framework to achieve an accurate representation of these highly complex supramolecular compounds.

Considering the pressing challenges associated with the rapidly accelerating climate change and the need for more efficient strategies to store and manipulate carbon dioxide and other technically relevant gases such as CH_4_ and H_2_, the highly favorable ratio between accuracy of results and computational effort in the presented DFTB MD simulation protocol is capable of bridging the gap between DFT and FF-based calculation approaches. While maintaining the many-body description inherent in a QM treatment of the electron density through appropriate parametrization of the orbital interactions, DFTB is about a factor of 100 to 1000 more efficient than DFT, enabling execution times per MD step in the range of 5–30 s. In addition, the application of constrained MD routines (i.e., the SHAKE/RATTLE algorithms) keeping the C–H bonds of the BDC linker units rigid does not influence the simulation approach in a negative way, while at the same time enabling an enlarged MD time step of 2.0 fs (i.e., a speed of factor of 4 to 10).

Nevertheless, a potential shortcoming of semiempirical methods is linked to the parametrization. In case the target system does not fall into a particular parametrization, the calculation is likely to result in inaccurate data when compared to experimental reference values. Thus, it is critical to assess the capabilities of a particular DFTB parametrization to verify that an adequate description of all properties of interest is achieved. In this work, it is demonstrated that the challenging properties of pristine MOF-5 (such as the thermal expansion coefficient and bulk modulus) as well as those of CO_2_@MOF-5 systems (interaction energy, self-diffusion coefficient) considering different loadings are in very good agreement with experimental results and other theoretical estimations. For this reason, it can be expected that DFTB MD simulations are capable of providing manifold insight into the physicochemical properties of a broad range of host–guest systems already in the near future.

## Supporting Information Available

Comparison of X-ray diffractogram of pristine MOF-5 and MSD of CO_2_ embedded in MOF-5 (PDF)

## References

[ref1] Lacis A. A.; Schmidt G. A.; Rind D.; Ruedy R. A. Atmospheric CO_2_: Principal Control Knob Governing Earth’s Temperature. Science 2010, 330, 356–359. 10.1126/science.1190653. 20947761

[ref2] Fawzy S.; Osman A. I.; Doran J.; Rooney D. W. Strategies for mitigation of climate change: a review. Environ. Chem. Lett. 2020, 18, 2069–2094. 10.1007/s10311-020-01059-w.

[ref3] United Nations Environment Programme. Emissions Gap Report; UNEP, 2019; p 80.

[ref4] Koutsonikolas D. E.; Pantoleontos G. T.; Kaldis S. P. In Current Trends and Future Developments on (Bio-) Membranes; Basile A., Favvas E. P., Eds.; Elsevier, 2018; pp 185–207.

[ref5] Sumida K.; Rogow D. L.; Mason J. A.; McDonald T. M.; Bloch E. D.; Herm Z. R.; Bae T.-H.; Long J. R. Carbon Dioxide Capture in Metal–Organic Frameworks. Chem. Rev. 2012, 112, 724–781. 10.1021/cr2003272. 22204561

[ref6] Millward A. R.; Yaghi O. M. Metal-Organic Frameworks with Exceptionally High Capacity for Storage of Carbon Dioxide at Room Temperature. J. Am. Chem. Soc. 2005, 127, 17998–17999. 10.1021/ja0570032. 16366539

[ref7] Babarao R.; Jiang J. Diffusion and Separation of CO_2_ and CH_4_ in Silicalite, C168 Schwarzite, and IRMOF-1: A Comparative Study from Molecular Dynamics Simulation. Langmuir 2008, 24, 5474–5484. 10.1021/la703434s. 18433152

[ref8] Freund R.; Zaremba O.; Arnauts G.; Ameloot R.; Skorupskii G.; Dincă M.; Bavykina A.; Gascon J.; Ejsmont A.; Goscianska J.; et al. The Current Status of MOF and COF Applications. Angew. Chem., Int. Ed. 2021, 60, 23975–24001. 10.1002/anie.202106259. 33989445

[ref9] Farha O. K.; Eryazici I.; Jeong N. C.; Hauser B. G.; Wilmer C. E.; Sarjeant A. A.; Snurr R. Q.; Nguyen S. T.; Yazaydın A. Ö.; Hupp J. T. Metal–Organic Framework Materials with Ultrahigh Surface Areas: Is the Sky the Limit?. J. Am. Chem. Soc. 2012, 134, 15016–15021. 10.1021/ja3055639. 22906112

[ref10] Bukowski B. C.; Keil F. J.; Ravikovitch P. I.; Sastre G.; Snurr R. Q.; Coppens M.-O. Connecting theory and simulation with experiment for the study of diffusion in nanoporous solids. Adsorption 2021, 27, 683–760. 10.1007/s10450-021-00314-y.

[ref11] Hu H.; Du L.; Xing Y.; Li X. Detailed study on self- and multicomponent diffusion of CO_2_-CH_4_ gas mixture in coal by molecular simulation. Fuel 2017, 187, 220–228. 10.1016/j.fuel.2016.09.056.

[ref12] Murray L. J.; Dincă M.; Long J. R. Hydrogen storage in metal–organic frameworks. Chem. Soc. Rev. 2009, 38, 1294–1314. 10.1039/b802256a. 19384439

[ref13] Zhao D.; Yuan D.; Zhou H.-C. The current status of hydrogen storage in metal–organic frameworks. Energy Environ. Sci. 2008, 1, 222–235. 10.1039/b808322n.

[ref14] Sculley J.; Yuan D.; Zhou H.-C. The current status of hydrogen storage in metal–organic frameworks updated. Energy Environ. Sci. 2011, 4, 2721–2735. 10.1039/c1ee01240a.

[ref15] Kumar P.; Pournara A.; Kim K.-H.; Bansal V.; Rapti S.; Manos M. J. Metal-organic frameworks: Challenges and opportunities for ion-exchange/sorption applications. Prog. Mater. Sci. 2017, 86, 25–74. 10.1016/j.pmatsci.2017.01.002.

[ref16] Rösler C.; Fischer R. A. Metal–organic frameworks as hosts for nanoparticles. CrystEngComm 2015, 17, 199–217. 10.1039/C4CE01251H.

[ref17] Moon H. R.; Lim D.-W.; Suh M. P. Fabrication of metal nanoparticles in metal–organic frameworks. Chem. Soc. Rev. 2013, 42, 1807–1824. 10.1039/C2CS35320B. 23192676

[ref18] Cai H.; Huang Y.-L.; Li D. Biological metal–organic frameworks: Structures, host–guest chemistry and bio-applications. Coord. Chem. Rev. 2019, 378, 207–221. 10.1016/j.ccr.2017.12.003.

[ref19] Ma S.; Zhou H.-C. Gas storage in porous metal–organic frameworks for clean energy applications. Chem. Commun. 2010, 46, 44–53. 10.1039/B916295J. 20024292

[ref20] Li H.; Eddaoudi M.; O’Keeffe M.; Yaghi O. M. Design and synthesis of an exceptionally stable and highly porous metal-organic framework. Nature 1999, 402, 276–279. 10.1038/46248.

[ref21] Wang J.-L.; Wang C.; Lin W. Metal–Organic Frameworks for Light Harvesting and Photocatalysis. ACS Catal. 2012, 2, 2630–2640. 10.1021/cs3005874.

[ref22] Corma A.; García H.; Llabrés i Xamena F. X. Engineering Metal Organic Frameworks for Heterogeneous Catalysis. Chem. Rev. 2010, 110, 4606–4655. 10.1021/cr9003924. 20359232

[ref23] Joarder B.; Desai A. V.; Samanta P.; Mukherjee S.; Ghosh S. K. Selective and Sensitive Aqueous-Phase Detection of 2,4,6-Trinitrophenol (TNP) by an Amine-Functionalized Metal–Organic Framework. Chem.—Eur. J. 2015, 21, 965–969. 10.1002/chem.201405167. 25424400

[ref24] Liu X.-G.; Wang H.; Chen B.; Zou Y.; Gu Z.-G.; Zhao Z.; Shen L. A luminescent metal–organic framework constructed using a tetraphenylethene-based ligand for sensing volatile organic compounds. Chem. Commun. 2015, 51, 1677–1680. 10.1039/C4CC08945F. 25502496

[ref25] Morozan A.; Jaouen F. Metal organic frameworks for electrochemical applications. Energy Environ. Sci. 2012, 5, 9269–9290. 10.1039/c2ee22989g.

[ref26] Cai M.; Qin L.; You L.; Yao Y.; Wu H.; Zhang Z.; Zhang L.; Yin X.; Ni J. Functionalization of MOF-5 with mono-substituents: effects on drug delivery behavior. RSC Adv. 2020, 10, 36862–36872. 10.1039/D0RA06106A. 35517920 PMC9057024

[ref27] He S.; Wu L.; Li X.; Sun H.; Xiong T.; Liu J.; Huang C.; Xu H.; Sun H.; Chen W.; et al. Metal-organic frameworks for advanced drug delivery. Acta Pharm. Sin. B 2021, 11, 2362–2395. 10.1016/j.apsb.2021.03.019. 34522591 PMC8424373

[ref28] Li H.; Li L.; Lin R.-B.; Zhou W.; Zhang Z.; Xiang S.; Chen B. Porous metal-organic frameworks for gas storage and separation: Status and challenges. EnergyChem 2019, 1, 100006 10.1016/j.enchem.2019.100006. PMC11071076 38711814

[ref29] Furukawa H.; Ko N.; Go Y. B.; Aratani N.; Choi S. B.; Choi E.; Yazaydin A. Ö.; Snurr R. Q.; O’Keeffe M.; Kim J.; et al. Ultrahigh Porosity in Metal-Organic Frameworks. Science 2010, 329, 424–428. 10.1126/science.1192160. 20595583

[ref30] Choi J.-S.; Son W.-J.; Kim J.; Ahn W.-S. Metal–organic framework MOF-5 prepared by microwave heating: Factors to be considered. Microporous Mesoporous Mater. 2008, 116, 727–731. 10.1016/j.micromeso.2008.04.033.

[ref31] Rosi N. L.; Eckert J.; Eddaoudi M.; Vodak D. T.; Kim J.; O’Keeffe M.; Yaghi O. M. Hydrogen Storage in Microporous Metal-Organic Frameworks. Science 2003, 300, 1127–1129. 10.1126/science.1083440. 12750515

[ref32] Kaye S. S.; Dailly A.; Yaghi O. M.; Long J. R. Impact of Preparation and Handling on the Hydrogen Storage Properties of Zn_4_O(1,4-benzenedicarboxylate)_3_ (MOF-5). J. Am. Chem. Soc. 2007, 129, 14176–14177. 10.1021/ja076877g. 17967030

[ref33] Li J.-R.; Sculley J.; Zhou H.-C. Metal–Organic Frameworks for Separations. Chem. Rev. 2012, 112, 869–932. 10.1021/cr200190s. 21978134

[ref34] Bristow J. K.; Tiana D.; Walsh A. Transferable Force Field for Metal–Organic Frameworks from First-Principles: BTW-FF. J. Chem. Theory Comput. 2014, 10, 4644–4652. 10.1021/ct500515h. 25574157 PMC4284133

[ref35] Forrest K. A.; Pham T.; McLaughlin K.; Belof J. L.; Stern A. C.; Zaworotko M. J.; Space B. Simulation of the Mechanism of Gas Sorption in a Metal–Organic Framework with Open Metal Sites: Molecular Hydrogen in PCN-61. J. Phys. Chem. C 2012, 116, 15538–15549. 10.1021/jp306084t.

[ref36] Mercado R.; Vlaisavljevich B.; Lin L.-C.; Lee K.; Lee Y.; Mason J. A.; Xiao D. J.; Gonzalez M. I.; Kapelewski M. T.; Neaton J. B.; et al. Force Field Development from Periodic Density Functional Theory Calculations for Gas Separation Applications Using Metal–Organic Frameworks. J. Phys. Chem. C 2016, 120, 12590–12604. 10.1021/acs.jpcc.6b03393.

[ref37] Becker T. Molecular simulation of tunable materials: Metal-organic frameworks ionic liquids theory application. Ph.D. Dissertation; Delft University of Technology, 2019; p 174.

[ref38] Qasem N. A.; Ben-Mansour R.; Habib M. A. An efficient CO_2_ adsorptive storage using MOF-5 and MOF-177. Applied Energy 2018, 210, 317–326. 10.1016/j.apenergy.2017.11.011.

[ref39] Saha D.; Bao Z.; Jia F.; Deng S. Adsorption of CO_2_, CH_4_, N_2_O, and N_2_ on MOF-5, MOF-177, and Zeolite 5A. Environ. Sci. Technol. 2010, 44, 1820–1826. 10.1021/es9032309. 20143826

[ref40] Marco-Lozar J.; Juan-Juan J.; Suárez-García F.; Cazorla-Amorós D.; Linares-Solano A. MOF-5 and activated carbons as adsorbents for gas storage. Int. J. Hydrogen Energy 2012, 37, 2370–2381. 10.1016/j.ijhydene.2011.11.023.

[ref41] Sun M.; Wang X.; Gao F.; Xu M.; Fan W.; Xu B.; Sun D. Synthesis strategies of metal-organic frameworks for CO_2_ capture. Microstructures 2023, 3, 2023032 10.20517/microstructures.2023.32.

[ref42] Zulys A.; Yulia F.; Muhadzib N.; Nasruddin Biological Metal–Organic Frameworks (Bio-MOFs) for CO_2_ Capture. Ind. Eng. Chem. Res. 2021, 60, 37–51. 10.1021/acs.iecr.0c04522.

[ref43] Skoulidas A. I. Molecular Dynamics Simulations of Gas Diffusion in Metal-Organic Frameworks: Argon in CuBTC. J. Am. Chem. Soc. 2004, 126, 1356–1357. 10.1021/ja039215+. 14759190

[ref44] Skoulidas A. I.; Sholl D. S. Self-Diffusion and Transport Diffusion of Light Gases in Metal-Organic Framework Materials Assessed Using Molecular Dynamics Simulations. J. Phys. Chem. B 2005, 109, 15760–15768. 10.1021/jp051771y. 16853000

[ref45] Yang Q.; Zhong C. Molecular Simulation of Adsorption and Diffusion of Hydrogen in Metal-Organic Frameworks. J. Phys. Chem. B 2005, 109, 11862–11864. 10.1021/jp051903n. 16852458

[ref46] Sarkisov L.; Düren T.; Snurr R. Q. Molecular modelling of adsorption in novel nanoporous metal–organic materials. Mol. Phys. 2004, 102, 211–221. 10.1080/00268970310001654854.

[ref47] Wu X.-P.; Choudhuri I.; Truhlar D. G. Computational Studies of Photocatalysis with Metal–Organic Frameworks. Energy Environ. Mater. 2019, 2, 251–263. 10.1002/eem2.12051.

[ref48] Lin L.-C.; Lee K.; Gagliardi L.; Neaton J. B.; Smit B. Force-Field Development from Electronic Structure Calculations with Periodic Boundary Conditions: Applications to Gaseous Adsorption and Transport in Metal–Organic Frameworks. J. Chem. Theory Comput. 2014, 10, 1477–1488. 10.1021/ct500094w. 26580364

[ref49] Elstner M.; Porezag D.; Jungnickel G.; Elsner J.; Haugk M.; Frauenheim T.; Suhai S.; Seifert G. Self-consistent-charge density-functional tight-binding method for simulations of complex materials properties. Phys. Rev. B: Condens. Matter Mater. Phys. 1998, 58, 7260–7268. 10.1103/PhysRevB.58.7260.

[ref50] Oliveira A. F.; Seifert G.; Heine T.; Duarte H. A. Density-Functional Based Tight-Binding: an Approximate DFT Method. J. Braz. Chem. Soc. 2009, 20, 1193–1205. 10.1590/s0103-50532009000700002.

[ref51] Maupin C. M.; Aradi B.; Voth G. A. The Self-Consistent Charge Density Functional Tight Binding Method Applied to Liquid Water and the Hydrated Excess Proton: Benchmark Simulations. J. Phys. Chem. B 2010, 114, 6922–6931. 10.1021/jp1010555. 20426461

[ref52] Lukose B.; Supronowicz B.; St Petkov P.; Frenzel J.; Kuc A. B.; Seifert G.; Vayssilov G. N.; Heine T. Structural properties of metal-organic frameworks within the density-functional based tight-binding method. Phys. Status Solidi B 2012, 249, 335–342. 10.1002/pssb.201100634.

[ref53] Zentel T.; Kühn O. Properties of hydrogen bonds in the protic ionic liquid ethylammonium nitrate. Theor. Chem. Acc. 2017, 136, 87 10.1007/s00214-017-2119-6.

[ref54] Purtscher F. R. S.; Christanell L.; Schulte M.; Seiwald S.; Rödl M.; Ober I.; Maruschka L. K.; Khoder H.; Schwartz H. A.; Bendeif E.-E.; et al. Structural Properties of Metal–Organic Frameworks at Elevated Thermal Conditions via a Combined Density Functional Tight Binding Molecular Dynamics (DFTB MD) Approach. J. Phys. Chem. C 2023, 127, 1560–1575. 10.1021/acs.jpcc.2c05103. PMC9884096 36721770

[ref55] Hofer T. S.; Listyarini R. V.; Hajdarevic E.; Maier L.; Purtscher F. R. S.; Gamper J.; Hanser F. Beyond the Status Quo: Density Functional Tight Binding and Neural Network Potentials as a Versatile Simulation Strategy to Characterize Host–Guest Interactions in Metal- and Covalent Organic Frameworks. J. Phys. Chem. Lett. 2023, 14, 6018–6027. 10.1021/acs.jpclett.3c00941. 37352552 PMC10331828

[ref56] Elstner M.; Frauenheim T.; Kaxiras E.; Seifert G.; Suhai S. A Self-Consistent Charge Density-Functional Based Tight-Binding Scheme for Large Biomolecules. Phys. Status Solidi B 2000, 217, 357–376. 10.1002/(SICI)1521-3951(200001)217:1<357::AID-PSSB357>3.0.CO;2-J.

[ref57] Elstner M.; Seifert G. Density functional tight binding. Philos. Trans. R. Soc., A 2014, 372, 20120483 10.1098/rsta.2012.0483. 24516180

[ref58] Seifert G.; Joswig J.-O. Density-functional tight binding an approximate density-functional theory method. Wiley Interdiscip. Rev.: Comput. Mol. Sci. 2012, 2, 456–465. 10.1002/wcms.1094.

[ref59] Koskinen P.; Mäkinen V. Density-functional tight-binding for beginners. Comput. Mater. Sci. 2009, 47, 237–253. 10.1016/j.commatsci.2009.07.013.

[ref60] Chadi D. J. Atomic and Electronic Structures of Reconstructed Si(100) Surfaces. Phys. Rev. Lett. 1979, 43, 43–47. 10.1103/PhysRevLett.43.43.

[ref61] Walter A. H. Electronic structure and the properties of solids: the physics of the chemical bond. J. Mol. Struct. 1980, 71, 355 10.1016/0022-2860(81)85136-8.

[ref62] Elstner M. SCC-DFTB: What Is the Proper Degree of Self-Consistency?. J. Phys. Chem. A 2007, 111, 5614–5621. 10.1021/jp071338j. 17564420

[ref63] Köhler C.; Seifert G.; Gerstmann U.; Elstner M.; Overhof H.; Frauenheim T. Approximate density-functional calculations of spin densities in large molecular systems and complex solids. Phys. Chem. Chem. Phys. 2001, 3, 5109–5114. 10.1039/b105782k.

[ref64] Gaus M.; Cui Q.; Elstner M. DFTB3: Extension of the Self-Consistent-Charge Density-Functional Tight-Binding Method (SCC-DFTB). J. Chem. Theory Comput. 2011, 7, 931–948. 10.1021/ct100684s. PMC3509502 23204947

[ref65] Gaus M.; Goez A.; Elstner M. Parametrization and Benchmark of DFTB3 for Organic Molecules. J. Chem. Theory Comput. 2013, 9, 338–354. 10.1021/ct300849w. 26589037

[ref66] Lu X.; Gaus M.; Elstner M.; Cui Q. Parametrization of DFTB3/3OB for Magnesium and Zinc for Chemical and Biological Applications. J. Phys. Chem. B 2015, 119, 1062–1082. 10.1021/jp506557r. 25178644 PMC4306495

[ref67] Grimme S. Density functional theory with London dispersion corrections. Wiley Interdiscip. Rev.: Comput. Mol. Sci. 2011, 1, 211–228. 10.1002/wcms.30.

[ref68] Grimme S.; Ehrlich S.; Goerigk L. Effect of the damping function in dispersion corrected density functional theory. J. Comput. Chem. 2011, 32, 1456–1465. 10.1002/jcc.21759. 21370243

[ref69] Caldeweyher E.; Mewes J.-M.; Ehlert S.; Grimme S. Extension and evaluation of the D4 London-dispersion model for periodic systems. Phys. Chem. Chem. Phys. 2020, 22, 8499–8512. 10.1039/D0CP00502A. 32292979

[ref70] Monkhorst H. J.; Pack J. D. Special points for Brillouin-zone integrations. Phys. Rev. B: Solid State 1976, 13, 5188–5192. 10.1103/PhysRevB.13.5188.

[ref71] Hofer T.; Pribil A.; Randolf B.; Rode M. Chapter 7 - Ab Initio Quantum Mechanical Charge Field Molecular Dynamics: A Nonparametrized First-Principle Approach to Liquids and Solutions. Adv. Quantum Chem. 2010, 59, 213–246. 10.1016/S0065-3276(10)59007-5.

[ref72] Rode B. M.; Hofer T. S.; Randolf B. R.; Schwenk C. F.; Xenides D.; Vchirawongkwin V. Ab initio quantum mechanical charge field (QMCF) molecular dynamics: a QM/MM – MD procedure for accurate simulations of ions and complexes. Theor. Chem. Acc. 2006, 115, 77–85. 10.1007/s00214-005-0049-1.

[ref73] Listyarini R. V.; Kriesche B. M.; Hofer T. S. The solvation structure of CO_2_ in dichloromethane – A comparative correlated, semi-empirical and classical MD simulation study. J. Mol. Liq. 2022, 363, 119840 10.1016/j.molliq.2022.119840.

[ref74] Hourahine B.; Aradi B.; Blum V.; Bonafé F.; Buccheri A.; Camacho C.; Cevallos C.; Deshaye M. Y.; Dumitrică T.; Dominguez A.; et al. DFTB+, a software package for efficient approximate density functional theory based atomistic simulations. J. Chem. Phys. 2020, 152, 124101 10.1063/1.5143190. 32241125

[ref75] Andersen H. C. Molecular dynamics simulations at constant pressure and/or temperature. J. Chem. Phys. 1980, 72, 2384–2393. 10.1063/1.439486.

[ref76] Ryckaert J.-P.; Ciccotti G.; Berendsen H. J. Numerical integration of the cartesian equations of motion of a system with constraints: molecular dynamics of n-alkanes. J. Comput. Phys. 1977, 23, 327–341. 10.1016/0021-9991(77)90098-5.

[ref77] Andersen H. C. Rattle: A “velocity” version of the shake algorithm for molecular dynamics calculations. Comput. Phys. 1983, 52, 24–34. 10.1016/0021-9991(83)90014-1.

[ref78] Berendsen H. J. C.; Postma J. P. M.; van Gunsteren W. F.; DiNola A.; Haak J. R. Molecular dynamics with coupling to an external bath. J. Chem. Phys. 1984, 81, 3684–3690. 10.1063/1.448118.

[ref79] Greathouse J. A.; Allendorf M. D. Force Field Validation for Molecular Dynamics Simulations of IRMOF-1 and Other Isoreticular Zinc Carboxylate Coordination Polymers. J. Phys. Chem. C 2008, 112, 5795–5802. 10.1021/jp076853w.

[ref80] Izumi F.; Momma K. Applied Crystallography XX; Trans Tech Publications Ltd, 2007; Vol. 130, pp 15–20.

[ref81] Momma K.; Izumi F. VESTA3 for three-dimensional visualization of crystal, volumetric and morphology data. J. Appl. Crystallogr. 2011, 44, 1272–1276. 10.1107/S0021889811038970.

[ref82] Rödl M.; Kerschbaumer S.; Kopacka H.; Blaser L.; Purtscher F. R. S.; Huppertz H.; Hofer T. S.; Schwartz H. A. Structural, dynamical, and photochemical properties of ortho-tetrafluoroazobenzene inside a flexible MOF under visible light irradiation. RSC Adv. 2021, 11, 3917–3930. 10.1039/D0RA10500G. 35424349 PMC8694203

[ref83] Einstein A. Über die von der molekularkinetischen Theorie der Wärme geforderte Bewegung von in ruhenden Flüssigkeiten suspendierten Teilchen. Ann. Phys. 1905, 322, 549–560. 10.1002/andp.19053220806.

[ref84] Sun Y.; Sun H. An all-atom force field developed for Zn_4_O(RCO_2_)_6_ metal organic frameworks. J. Mol. Model. 2014, 20, 2146 10.1007/s00894-014-2146-3. 24562858

[ref85] Stallmach F.; Gröger S.; Künzel V.; Kärger J.; Yaghi O. M.; Hesse M.; Müller U. NMR Studies on the Diffusion of Hydrocarbons on the Metal-Organic Framework Material MOF-5. Angew. Chem., Int. Ed. 2006, 45, 2123–2126. 10.1002/anie.200502553. 16498688

[ref86] Yang J.; Grzech A.; Mulder F. M.; Dingemans T. J. The hydrogen storage capacity of mono-substituted MOF-5 derivatives: An experimental and computational approach. Microporous Mesoporous Mater. 2013, 171, 65–71. 10.1016/j.micromeso.2012.12.035.

[ref87] Zhang Z.; Huang S.; Xian S.; Xi H.; Li Z. Adsorption Equilibrium and Kinetics of CO_2_ on Chromium Terephthalate MIL-101. Energy Fuels 2011, 25, 835–842. 10.1021/ef101548g.

[ref88] Zhou W.; Wu H.; Yildirim T.; Simpson J. R.; Walker A. R. H. Origin of the exceptional negative thermal expansion in metal-organic framework-5 Zn_4_O(1,4 – benzenedicarboxylate)_3_. Phys. Rev. B: Condens. Matter Mater. Phys. 2008, 78, 054114 10.1103/PhysRevB.78.054114.

[ref89] Ryder M. R.; Maul J.; Civalleri B.; Erba A. Quasi-Harmonic Lattice Dynamics of a Prototypical Metal–Organic Framework. Adv. Theory Simul. 2019, 2, 1900093 10.1002/adts.201900093.

[ref90] Lock N.; Wu Y.; Christensen M.; Cameron L. J.; Peterson V. K.; Bridgeman A. J.; Kepert C. J.; Iversen B. B. Elucidating Negative Thermal Expansion in MOF-5. J. Phys. Chem. C 2010, 114, 16181–16186. 10.1021/jp103212z.

[ref91] Lock N.; Christensen M.; Wu Y.; Peterson V. K.; Thomsen M. K.; Piltz R. O.; Ramirez-Cuesta A. J.; McIntyre G. J.; Norén K.; Kutteh R.; et al. Scrutinizing negative thermal expansion in MOF-5 by scattering techniques and ab initio calculations. Dalton Trans. 2013, 42, 1996–2007. 10.1039/C2DT31491F. 23044752

[ref92] Wieme J.; Van Speybroeck V. Unravelling thermal stress due to thermal expansion mismatch in metal–organic frameworks for methane storage. J. Mater. Chem. A 2021, 9, 4898–4906. 10.1039/D0TA09462E.

[ref93] Wieme J.; Vandenbrande S.; Lamaire A.; Kapil V.; Vanduyfhuys L.; Van Speybroeck V. Thermal Engineering of Metal–Organic Frameworks for Adsorption Applications: A Molecular Simulation Perspective. ACS Appl. Mater. Interfaces 2019, 11, 38697–38707. 10.1021/acsami.9b12533. 31556593 PMC6818952

[ref94] Dubbeldam D.; Walton K.; Ellis D.; Snurr R. Exceptional Negative Thermal Expansion in Isoreticular Metal–Organic Frameworks. Angew. Chem., Int. Ed. 2007, 46, 4496–4499. 10.1002/anie.200700218. 17487904

[ref95] Boyd P. G.; Moosavi S. M.; Witman M.; Smit B. Force-Field Prediction of Materials Properties in Metal-Organic Frameworks. J. Phys. Chem. Lett. 2017, 8, 357–363. 10.1021/acs.jpclett.6b02532. 28008758 PMC5253710

[ref96] Lamaire A.; Wieme J.; Rogge S. M. J.; Waroquier M.; Van Speybroeck V. On the importance of anharmonicities and nuclear quantum effects in modelling the structural properties and thermal expansion of MOF-5. J. Chem. Phys. 2019, 150, 094503 10.1063/1.5085649. 30849909

[ref97] Wang L.; Wang C.; Sun Y.; Shi K.; Deng S.; Lu H. Large negative thermal expansion provided by metal-organic framework MOF-5: A first-principles study. Mater. Chem. Phys. 2016, 175, 138–145. 10.1016/j.matchemphys.2016.03.003.

[ref98] Dove M. T.; Fang H. Negative thermal expansion and associated anomalous physical properties: review of the lattice dynamics theoretical foundation. Rep. Prog. Phys. 2016, 79, 066503 10.1088/0034-4885/79/6/066503. 27177210

[ref99] Chen J.; Hu L.; Deng J.; Xing X. Negative thermal expansion in functional materials: controllable thermal expansion by chemical modifications. Chem. Soc. Rev. 2015, 44, 3522–3567. 10.1039/C4CS00461B. 25864730

[ref100] Burgaz E.; Erciyes A.; Andac M.; Andac O. Synthesis and characterization of nano-sized metal organic framework-5 (MOF-5) by using consecutive combination of ultrasound and microwave irradiation methods. Inorg. Chim. Acta 2019, 485, 118–124. 10.1016/j.ica.2018.10.014.

[ref101] Eddaoudi M.; Kim J.; Rosi N.; Vodak D.; Wachter J.; O’Keeffe M.; Yaghi O. M. Systematic Design of Pore Size and Functionality in Isoreticular MOFs and Their Application in Methane Storage. Science 2002, 295, 469–472. 10.1126/science.1067208. 11799235

[ref102] Banlusan K.; Strachan A. First-principles study of elastic mechanical responses to applied deformation of metal-organic frameworks. J. Chem. Phys. 2017, 146, 184705 10.1063/1.4982356.

[ref103] Farrusseng D.; Daniel C.; Gaudillère C.; Ravon U.; Schuurman Y.; Mirodatos C.; Dubbeldam D.; Frost H.; Snurr R. Q. Heats of Adsorption for Seven Gases in Three Metal-Organic Frameworks: Systematic Comparison of Experiment and Simulation. Langmuir 2009, 25, 7383–7388. 10.1021/la900283t. 19496548

[ref104] Jung J. Y.; Karadas F.; Zulfiqar S.; Deniz E.; Aparicio S.; Atilhan M.; Yavuz C. T.; Han S. M. Limitations and high pressure behavior of MOF-5 for CO_2_ capture. Phys. Chem. Chem. Phys. 2013, 15, 14319–14327. 10.1039/c3cp51768c. 23877231

[ref105] Yang J.; Grzech A.; Mulder F. M.; Dingemans T. J. Methyl modified MOF-5: a water stable hydrogen storage material. Chem. Commun. 2011, 47, 5244–5246. 10.1039/c1cc11054c. 21451855

[ref106] Tranchemontagne D. J.; Hunt J. R.; Yaghi O. M. Room temperature synthesis of metal-organic frameworks: MOF-5, MOF-74, MOF-177, MOF-199, and IRMOF-0. Tetrahedron 2008, 64, 8553–8557. 10.1016/j.tet.2008.06.036.

[ref107] Wang S.; Xie X.; Xia W.; Cui J.; Zhang S.; Du X. Study on the structure activity relationship of the crystal MOF-5 synthesis, thermal stability and N_2_ adsorption property. High Temp. Mater. Processes 2020, 39, 171–177. 10.1515/htmp-2020-0034.

[ref108] Mohamed S. A.; Chong S.; Kim J. Thermal Stability of Methyl-Functionalized MOF-5. J. Phys. Chem. C 2019, 123, 29686–29692. 10.1021/acs.jpcc.9b08060.

[ref109] Zhao Z.; Li Z.; Lin Y. S. Adsorption and Diffusion of Carbon Dioxide on Metal-Organic Framework (MOF-5). Ind. Eng. Chem. Res. 2009, 48, 10015–10020. 10.1021/ie900665f.

[ref110] Thissen P.; Wohlgemuth J.; Weidler P.; Smilgies D.; Heinke L.; Schewe N.; Koenig M.; Krolla P.; Wöll C. Elimination of Domain Boundaries Accelerates Diffusion in MOFs by an Order of Magnitude: Monolithic Metal-Organic Framework Thin Films Epitaxially Grown on Si(111) Substrates. Adv. Funct. Mater. 2023, 2301535 10.1002/adfm.202301535.

[ref111] Kriesche B. M.; Kronenberg L. E.; Purtscher F. R. S.; Hofer T. S. Storage and diffusion of CO_2_ in covalent organic frameworks A neural network-based molecular dynamics simulation approach. Front. Chem. 2023, 11, 1100210 10.3389/fchem.2023.1100210. 36970402 PMC10033539

[ref112] Tunca C.; Ford D. M. A transition-state theory approach to adsorbate dynamics at arbitrary loadings. J. Chem. Phys. 1999, 111, 2751–2760. 10.1063/1.479552.

[ref113] Tunca C.; Ford D. M. Modeling Cage-to-Cage Dynamics of Adsorbates at Arbitrary Loadings with Dynamically Corrected Transition-State Theory. J. Phys. Chem. B 2002, 106, 10982–10990. 10.1021/jp026375j.

[ref114] Skoulidas A. I.; Sholl D. S. Transport Diffusivities of CH_4_, CF_4_, He, Ne, Ar, Xe, and SF_6_ in Silicalite from Atomistic Simulations. J. Phys. Chem. B 2002, 106, 5058–5067. 10.1021/jp014279x.

[ref115] Yang Q.; Zhong C.; Chen J.-F. Computational Study of CO_2_ Storage in Metal-Organic Frameworks. J. Phys. Chem. C 2008, 112, 1562–1569. 10.1021/jp077387d.

[ref116] Salles F.; Jobic H.; Devic T.; Llewellyn P. L.; Serre C.; Férey G.; Maurin G. Self and Transport Diffusivity of CO_2_ in the Metal-Organic Framework MIL-47(V) Explored by Quasi-elastic Neutron Scattering Experiments and Molecular Dynamics Simulations. ACS Nano 2010, 4, 143–152. 10.1021/nn901132k. 19957953

[ref117] Afrin S.; Khan M. W.; Haque E.; Ren B.; Ou J. Z. Recent advances in the tuning of the organic framework materials – The selections of ligands, reaction conditions, and post-synthesis approaches. J. Colloid Interface Sci. 2022, 623, 378–404. 10.1016/j.jcis.2022.05.026. 35594596

[ref118] Koh K.; Wong-Foy A.; Matzger A. A Crystalline Mesoporous Coordination Copolymer with High Microporosity. Angew. Chem., Int. Ed. 2008, 47, 677–680. 10.1002/anie.200705020. 18058972

[ref119] Tafipolsky M.; Amirjalayer S.; Schmid R. Ab initio parametrized MM3 force field for the metal-organic framework MOF-5. J. Comput. Chem. 2007, 28, 1169–1176. 10.1002/jcc.20648. 17301955

[ref120] Addicoat M. A.; Vankova N.; Akter I. F.; Heine T. Extension of the Universal Force Field to Metal–Organic Frameworks. J. Chem. Theory Comput. 2014, 10, 880–891. 10.1021/ct400952t. 26580059

[ref121] Mattesini M.; Soler J. M.; Ynduráin F. Ab initio study of metal-organic framework-5 Zn_4_O(1,4–benzenedicarboxylate)_3_: An assessment of mechanical and spectroscopic properties. Phys. Rev. B: Condens. Matter Mater. Phys. 2006, 73, 094111 10.1103/PhysRevB.73.094111.

[ref122] Sagara T.; Klassen J.; Ganz E. Computational study of hydrogen binding by metal-organic framework-5. J. Chem. Phys. 2004, 121, 12543–12547. 10.1063/1.1809608. 15606275

[ref123] Civalleri B.; Napoli F.; Noël Y.; Roetti C.; Dovesi R. Ab-initio prediction of materials properties with CRYSTAL: MOF-5 as a case study. CrystEngComm 2006, 8, 364–371. 10.1039/B603150C.

[ref124] Yang L.-M.; Vajeeston P.; Ravindran P.; Fjellvåg H.; Tilset M. Theoretical Investigations on the Chemical Bonding, Electronic Structure, And Optical Properties of the Metal-Organic Framework MOF-5. Inorg. Chem. 2010, 49, 10283–10290. 10.1021/ic100694w. 20961146

[ref125] Bahr D. F.; Reid J. A.; Mook W. M.; Bauer C. A.; Stumpf R.; Skulan A. J.; Moody N. R.; Simmons B. A.; Shindel M. M.; Allendorf M. D. Mechanical properties of cubic zinc carboxylate IRMOF-1 metal-organic framework crystals. Phys. Rev. B: Condens. Matter Mater. Phys. 2007, 76, 184106 10.1103/PhysRevB.76.184106.

[ref126] Fuentes-Cabrera M.; Nicholson D. M.; Sumpter B. G.; Widom M. Electronic structure and properties of isoreticular metal-organic frameworks: The case of M-IRMOF1 (M = Zn, Cd, Be, Mg, and Ca). J. Chem. Phys. 2005, 123, 124713 10.1063/1.2037587. 16392517

[ref127] Wu H.; Yildirim T.; Zhou W. Exceptional Mechanical Stability of Highly Porous Zirconium Metal–Organic Framework UiO-66 and Its Important Implications. J. Phys. Chem. Lett. 2013, 4, 925–930. 10.1021/jz4002345. 26291357

[ref128] Samanta A.; Furuta T.; Li J. Theoretical assessment of the elastic constants and hydrogen storage capacity of some metal-organic framework materials. J. Chem. Phys. 2006, 125, 084714 10.1063/1.2337287. 16965046

