# Supplementary material for: Storage and Diffusion of Carbon Dioxide in the Metal Organic Framework MOF-5—A Semi-empirical Molecular Dynamics Study
Source: J Phys Chem B. 2023 Oct 19;127(43):9378–89. doi: 10.1021/acs.jpcb.3c04155 (PMC10627117; doi:10.1021/acs.jpcb.3c04155)
Supplement: Supplementary file 1 — jp3c04155_si_001.pdf [file jp3c04155_si_001.pdf]

Storage and Diffusion of Carbon Dioxide in the Metal Organic Framework MOF-5 - a Semi-Empirical Molecular Dynamics Study

Supporting Information

Risnita Vicky Listyarini[a,b], Jakob Gamper[a], Thomas S. Hofer[a,*]

[a] Theoretical Chemistry Division, Institute of General, Inorganic and Theoretical Chemistry, University of Innsbruck, Innrain 80-82A, A-6020 Innsbruck, Austria

[b] Chemistry Education Study Program, Sanata Dharma University, Yogyakarta 55282, Indonesia

[*] Corresponding author Email address: t.hofer@uibk.ac.at (T. S. Hofer)

1 Supporting Information

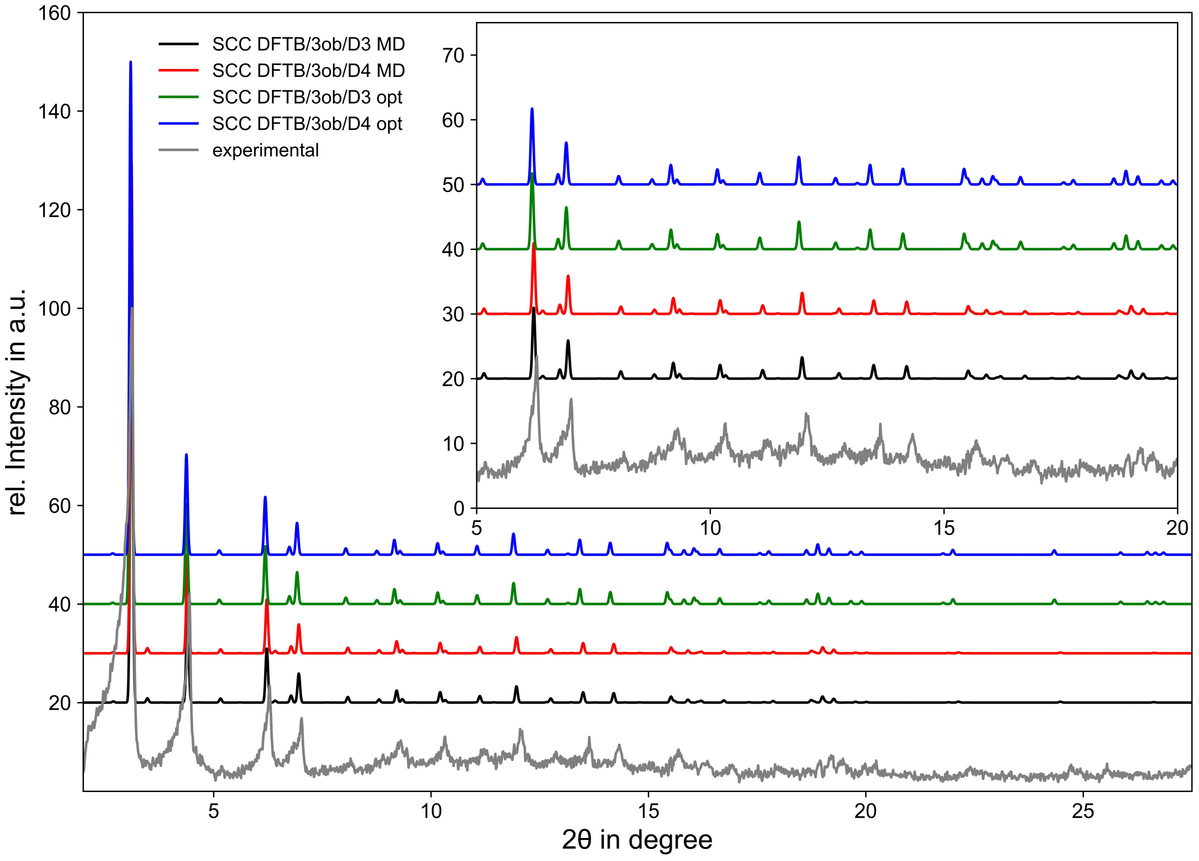

Figure S1: Comparison of X-ray diffractogram of MOF-5 via averaging over the MD trajectory at SCC DFTB/3ob/D3 (black) and SCC DFTB/3ob/D4 (red) and the associated optimised system at SCC DFTB/3ob/D3 (green) and and SCC DFTB/3ob/D4 (blue) and the experimental PXRD reference [1] (Mo K_α, $\lambda = 0.709319$ nm).

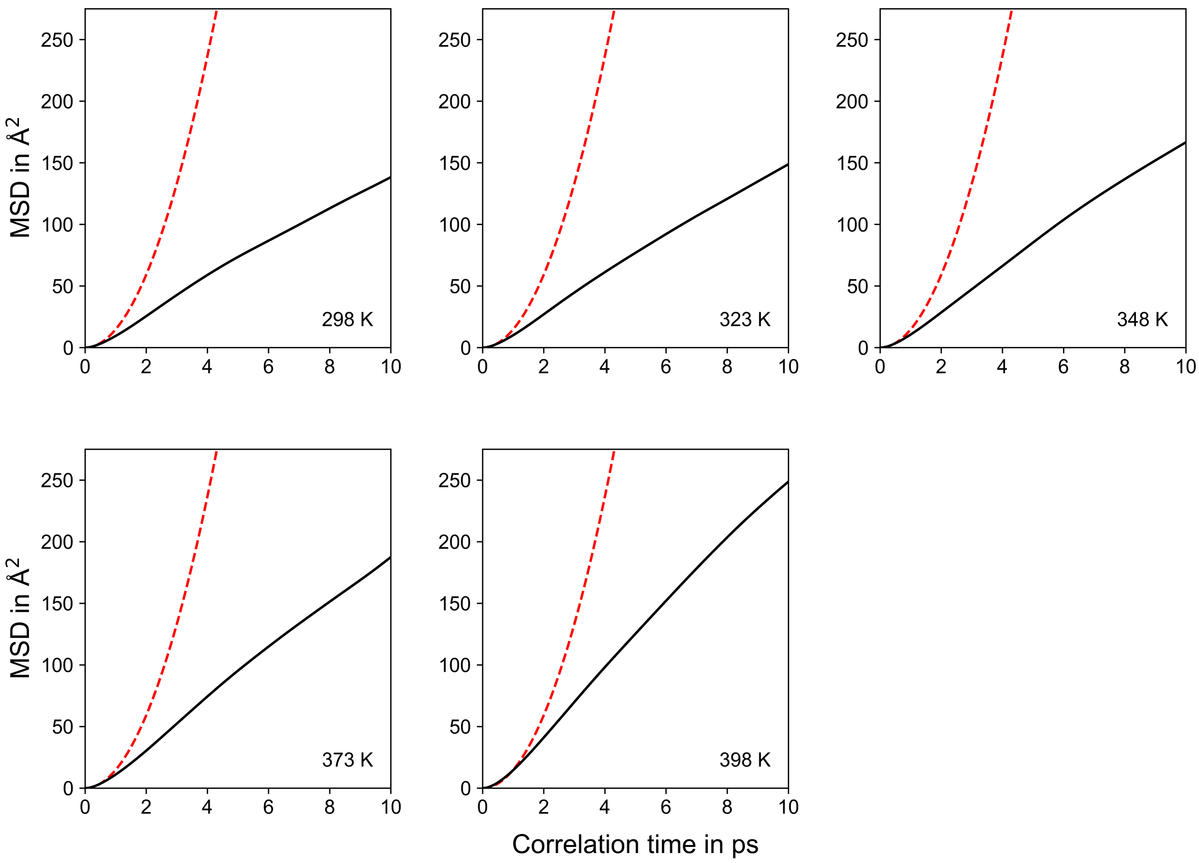

Figure S2: Mean square displacement (MSD) of CO_2 embedded in MOF-5 using correlation window of 10 ps. It can be seen that in all cases the linear diffusive regime is well separated from the corresponding ballistic region (red like) determined via a fit of a square function in the range of 0 to 0.7 ps.

References

(1) Purtscher, F. R. S.; Christanell, L.; Schulte, M.; Seiwald, S.; Rödl, M.; Ober, I.; Maruschka, L. K.; Khoder, H.; Schwartz, H. A.; Bendeif, E.-E.; Hofer, T. S. Structural Properties of Metal–Organic Frameworks at Elevated Thermal Conditions via a Combined Density Functional Tight Binding Molecular Dynamics (DFTB MD) Approach. *J. Phys. Chem. C* . **2023**, *127*, 1560–1575.
